# Announcing the 2018 *Toxics* Travel Award for Post-Doctoral Fellows

**DOI:** 10.3390/toxics6010010

**Published:** 2018-01-30

**Authors:** David Bellinger

**Affiliations:** Boston Children’s Hospital, Harvard Medical School, Harvard University, 300 Longwood Ave, Boston, MA 02115, USA; david.bellinger@childrens.harvard.edu

This year we enjoyed a large number of very highly meritorious applications for our annual *Toxics Travel Award*. It was not an easy task to select the top candidate. Nevertheless, with the assistance of our editorial board members, we have identified one outstanding candidate. Thus, as Editor-in-Chief of *Toxics*, I am pleased to announce the winner of the *Toxics Travel Award* for 2018 was granted to Dr. Joaquim Rovira, a post-doctoral researcher in Dr. Marta Schuhmacher’s lab at Rovira i Virgili University, Spain (Figure 1).

Born on 28 November 1981 in Reus, Spain, Joaquim Rovira is a graduate in Chemistry and Biochemistry (2004 and 2005, respectively), and obtained his PhD degree on March 2013.His thesis was entitled “Impact on human health of the use of alternative fuels in cement factories”. Currently, he is a post-doctoral researcher in the Environmental, Food, and Toxicological Technology Centre (TecnATox) at “Pere Virgili” Institute of Health Research (IISPV) and “Rovira i Virgili” University (URV), both located in Spain, in the laboratory of Prof. Marta Schuhmacher. Dr Rovira’s main areas of research are: Environmental and human (bio)monitoring of heavy metals, persistent organic pollutants (POPs) and endocrine disruptors; exposure and human health risk assessment; and optimization of monitoring methods through active and passive sampling of atmospheric contaminants (heavy metals and POPs). He has co-authored 26 papers in international peer-reviewed journals, and four book chapters and is supervising two pre-doctoral theses. He has been involved in several national and European projects: SCARCE, RISKCYCLE, TDS-Exposure, HBM4EU and HEALS. Currently, Dr Rovira is involved in several research projects one of them regarding prenatal exposure to endocrine disruptors in a cohort of pregnant women in a reference hospital in the region. Thanks to the *Toxics Travel award*, this work will be presented in the Society of Environmental Toxicology and Chemistry (SETAC) 28th annual meeting conference held in Rome between 13 and 17 May 2018.

The editors, managing editor and editorial board members join me in congratulating Dr. Joaquim Rovira on winning 2018 *Toxics Travel Award*. *Toxics* is proud to support Dr. Joaquim Rovira working in the field of toxicology and wish him further success in his career. We are grateful to all who submitted applications—thank you for letting us get to know you and your work. The future of toxicology looks very bright indeed. Finally, we are grateful to MDPI for their generous support of young scholars, helping them to share their work on the international stage.

## Figures and Tables

**Figure 1:**
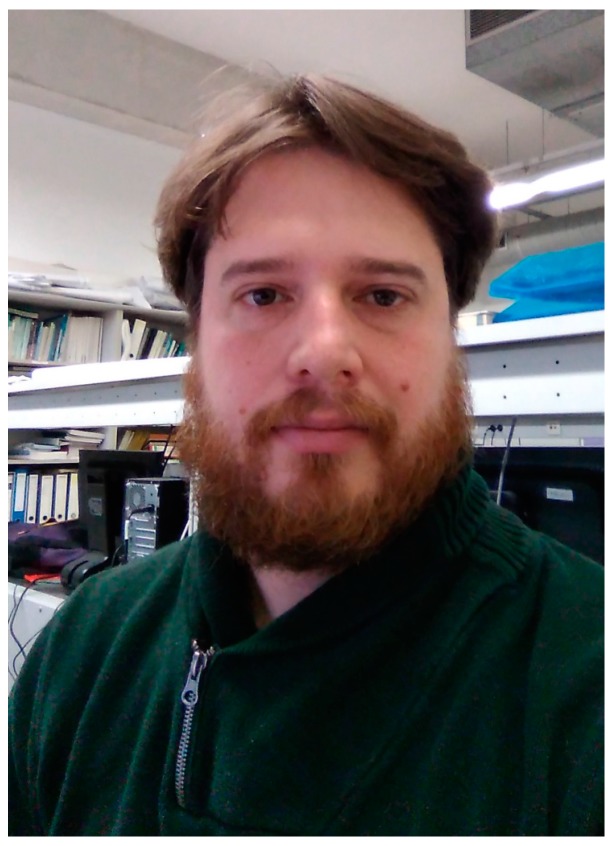
Dr. Joaquim Rovira.

